# Antioxidant and cytotoxic activity of propolis of *Plebeia droryana* and *Apis mellifera* (Hymenoptera, Apidae) from the Brazilian Cerrado biome

**DOI:** 10.1371/journal.pone.0183983

**Published:** 2017-09-12

**Authors:** Thaliny Bonamigo, Jaqueline Ferreira Campos, Alex Santos Oliveira, Heron Fernandes Vieira Torquato, José Benedito Perrella Balestieri, Claudia Andrea Lima Cardoso, Edgar Julian Paredes-Gamero, Kely de Picoli Souza, Edson Lucas dos Santos

**Affiliations:** 1 Research Group on Biotechnology and Bioprospecting Applied to Metabolism (GEBBAM), Federal University of Grande Dourados, Dourados, MS, Brazil; 2 Faculty of Pharmacy, Braz Cubas University, Francisco Rodrigues Filho Avenue, Mogi das Cruzes, SP, Brazil; 3 Department of Biochemistry, Federal University of São Paulo, R. Três de Maio 100, São Paulo, SP, Brazil; 4 Course of Chemistry, State University of Mato Grosso do Sul, Dourados, MS, Brazil; 5 Center for Interdisciplinary Research Biochemistry, University of Mogi das Cruzes, Av. Dr. Cândido Xavier de Almeida Souza, Mogi das Cruzes, SP, Brazil; University of British Columbia, CANADA

## Abstract

Propolis is a complex bioactive mixture produced by bees, known to have different biological activities, especially in countries where there is a rich biodiversity of plant species. The objective of this study was to determine the chemical composition and evaluate the antioxidant and cytotoxic properties of Brazilian propolis from the species *Plebeia droryana* and *Apis mellifera* found in Mato Grosso do Sul, Brazil. In the ethanolic extracts of *P*. *droryana* propolis (ExEP-P) and *A*. *mellifera* (ExEP-A) acids, phenolic compounds, terpenes and tocopherol were identified as major compounds. Both extracts presented antioxidant activity against the 2,2-diphenyl-1-picrylhydrazyl (DPPH) free radical, the maximum activities being 500 μg/mL (ExEP-P) and 300 μg/mL (ExEP-A). However, only ExEP-A was able to inhibit lipid peroxidation induced by the oxidizing agent 2,2'-azobis(2-amidinopropane) dihydrochloride (AAPH), which inhibited oxidative hemolysis and reduced the levels of malondialdehyde (MDA) in human erythrocytes for 4 h of incubation. The extracts also reduced the cell viability of the K562 erythroleukemia tumour line, with a predominance of necrotic death. Thus, it is concluded that the propolis produced by *P*. *droryana* and *A*. *mellifera* contain important compounds capable of minimizing the action of oxidizing substances in the organism and reducing the viability of erythroleukemia cells.

## Introduction

Propolis is a bee product resulting from the collection of resin from different parts of plants, such as buds of leaves, branches, flowers and pollen, with the addition of mandibular secretions from bees. Many bee species are capable of producing propolis, among them *Apis mellifera* [1] and some species of stingless bees known as meliponine bees [2,3]. In the hive, this resin is used to repair cracks or damage, to defend against microorganisms and to mummify the dead bodies of invading insects, preventing their decomposition and the contamination of the hive by fungi and bacteria [4,5].

Propolis is a complex mixture known to exhibit great chemical diversity, especially in tropical climate countries, where the richness of plant species is responsible for the presence of a wide variety of substances in propolis, such as phenolic compounds, flavonoids and terpenes [6,7]. However, the chemical composition of propolis depends on factors such as botanical origin, temperature variation and seasonality, as well as the salivary secretions and enzymes added to propolis by bees [5,8]. These changes can qualitatively and quantitatively alter the compounds, modifying their therapeutic properties [5,8,9].

Thus, propolis produced by different species of bees that cohabit the same region can present different biological substances and activities. Propolis from different parts of the world has been reported to have antioxidant [10,11], antibiofilm [12,13], antimicrobial [14–16], anti-inflammatory [17–19] and antitumour [20–22] activities.

For this reason, this bee product is of great interest to the pharmaceutical and food industries [23]. Studies have been conducted on propolis produced by different species of bees, to evaluate their chemical composition and their potential pharmacological activities [24,25].

The species of stingless bee *Plebeia droryana*, belonging to the subfamily Meliponinae, native to Brazil, Paraguay, Uruguay and Bolivia, is popularly known as the Mirim bee and produces a viscous propolis [3]. There are few studies on the existing compounds and biological activities of this product. Sawaya et al. [26] report some species of medicinal plants in which these bees collect resin for the production of propolis, such as *Schinus terebinthifolius* Raddi. In addition, studies with *P*. *droryana* propolis from the southeastern region of Brazil show that this product includes phenolic compounds and terpenes in its composition [27].

The species *Apis mellifera*, belonging to the subfamily Apinae, known as the European honey bee, is exotic in Brazil and is a major producer of propolis, which has been reported to present important antioxidant [28], antimicrobial [14,29] and antitumour [30,31] activities.

Thus, this study aimed to determine the chemical composition and the antioxidant and cytotoxic properties of the propolis of *P*. *droryana* and *A*. *mellifera* found in the Cerrado biome, in the Midwest region of Brazil.

## Materials and methods

### Ethics of experimentation

No specific permits were required for the described field studies. All field work to collect the propolis samples was conducted on private land and with owner permission. The field studies did not involve endangered or protected species. The protocol to collect human peripheral blood was approved by the Research Ethics Committee (Comitê de Ética em Pesquisa; CEP) of the University Center of Grande Dourados (Centro Universitário da Grande Dourados; UNIGRAN), Brazil (CEP process number 123/12). All subjects provided written informed consent for participation.

### Preparation of the ethanol extract of propolis (ExEP)

Propolis samples were collected from *P*. *droryana* and *A*. *mellifera* in the state of Mato Grosso do Sul, in the Midwest region of Brazil (22° 13’ 12” S—54° 49’ 2” W). For this, the identity of the bees species were authenticated by entomologist Professor José Benedito Perrella Balestieri, and four sample of propolis were collected in different seasons of the year of 2015, totalling 12.02 g (*P*. *droryana*) and 21.27 g (*A*. *mellifera*) of samples for each specie.

Ethanol extracts of propolis (ExEP) were prepared in the proportion of 4.5 mL of 80% ethanol per g of propolis. This solution was maintained at 70°C in a closed container in a water bath until complete dissolution and then filtered on 80 g/m^2^ qualitative filter paper (Prolab, São Paulo, Brazil) to obtain the ethanolic extract of propolis of *P*. *droryana* (ExEP- P) and *A*. *mellifera* (ExEP-A) [32]. After the extracts were prepared, they were identified, stored in closed containers and kept at -20°C until analysis.

### Chemical analysis

#### Preparation of the samples

The samples (1 mg) was fractionated with hexane and water in proportion 1:1 v:v and fraction soluble in hexane was analyzed by GC-MS and fraction soluble in water by HPLC. In addition, the GC-MS technique was employed to analyze highly volatilizable compounds that by the detector employed in this HPLC study would not be detected in the analysis.

#### GC-MS

Samples were injected and analyzed by gas chromatography-mass spectrometry (GC-MS). The GC-MS analysis was performed on a gas chromatograph (GC-2010 Plus, Shimadzu, Kyoto, Japan) equipped with a mass spectrometer detector (GC-MS Ultra 2010) using LM-5 (5% phenyl dimethylpolysiloxane) capillary column (15 m length × 0.2 mm i.d. and 0.2 μm film thickness) with initial oven temperature set at 150°C and heating from 150°C to 280°C at 15°C min^−1^ and a hold at 280°C for 15 min. Carrier gas of helium (99.999% and flow rate 1.0 mL min^−1^), 1 μL injection volume, split ratio (1:20). The injector temperature was 280°C and the quadrupole detector temperature was 280°C. The MS scan parameters included an electron-impact ionization voltage of 70 eV mass range of 45–600 *m/z* and scan interval of 0.3 s. The identifications were completed by comparing the mass spectra obtained in the NIST21 and WILEY229 libraries. In some cases, the compound was confirmed by comparison of standards. The standards from Sigma-Aldrich with purity ≥ 97%. Standards of the stigmasterol, β-sitosterol, β-amyrin, α-amyrin, β-amyrin acetate, α-amyrin acetate, tocopherol were prepared in the concentration initial of 1 mg/mL. The concentrations of compounds were determined by extern calibration after dilutions appropriated in the range of 0.1–50 μg/mL. The quantification of campesterol and taraxasterol were performed in relation to stigmasterol. All the samples were previously filtrated in 0.45 μm (Millex® Syringe Filter Millipore, Merck). The analysis was performed in triplicate.

#### HPLC

The extracts were analyzed in an analytical HPLC (LC-6AD, Shimadzu, Kyoto, Japan) system with a diode array detector (DAD) monitored at λ = 200–600 nm. The HPLC column was a C-18 (25 cm x 4.6 mm; particle size, 5 μm; Luna, Phenomenex, Torrance, CA, USA), with a small pre-column (2.5 cm x 3 mm) containing the same packing, used to protect the analytical column. In each analysis, the flow rate and the injected volume were set as 1.0 mL min^-1^ and 20 μL, respectively. All chromatographic analyses were performed at 22°C. Elution was carried out using a binary mobile phase of water with 6% acetic acid and 2 mM sodium acetate (eluent A) and acetonitrile (eluent B). The following applied gradient: 5% B (0 min), 15% B (30 min), 50% B (35 min) and 100% B (45 min). Standards of the vanillic acid, *p*-methylbenzoic acid, caffeic acid, ferrulic acid, *p*-coumaric acid, benzoic acid, cinnamic acid, rutin, sinapic acid, quercetin, luteolin, apigenin and vanilline (Sigma, ≥ 97%) were prepared in the concentration initial of 1 mg/mL. The concentrations of compounds were determined by extern calibration after dilutions appropriated in the range of 0.01–10 μg/mL. All the samples were previously filtrated in 0.45 μm (Millex® Syringe Filter Millipore, Merck). The analysis was performed in triplicate.

### Antioxidant activity

#### Free radical-scavenger activity

Free radical-scavenger activity was determined by the 2,2-diphenyl-1-picrylhydrazyl (DPPH) assay, as described previously by Gupta and Gupta [33], with some modifications. The antiradical activity of the extracts was evaluated using a dilution series, which involved the mixing of 1.8 mL of DPPH solution (0.11 mM DPPH in 80% ethanol) with 0.2 mL of ExEP-P or ExEP-A (1–500 μg/mL). After 30 min, the remaining DPPH radicals were quantified by absorption at 517 nm. The absorbance of each concentration of the ExEP (only sample with 80% ethanol) was subtracted from absorbance of the samples with DPPH solution. Ascorbic acid and butylated hydroxytoluene (BHT) were used as reference antioxidants. The tests were performed in duplicate in three independent experiments. DPPH solution without the tested sample was used as a control. The percentage inhibition was calculated from the control with the following Eq 1:

Scavengingactivity(%)=(1−Abssample/Abscontrol)x100 (1)

### Protection against lipid peroxidation using a human erythrocyte model

#### Preparation of erythrocyte suspensions

Following approval by the Research Ethics Committee, 20 mL samples of peripheral blood were collected from healthy donors into sodium citrate-containing tubes and subsequently centrifuged at 1500 rpm for 10 min. After centrifugation, the blood plasma and leukocyte layers were discarded, and the erythrocytes were washed three times with 0.9% sodium chloride solution (NaCl) and centrifuged at 1500 rpm for 10 min. Finally, 10% erythrocyte suspensions were prepared in 0.9% NaCl.

#### Oxidative hemolysis inhibition assay

The antioxidant activity in biological model was evaluated using human erythrocytes subjected to hemolysis via the oxidation of lipids and proteins of the cell membranes by the action of peroxyl free radicals generated by the oxidizing agent AAPH. The protective effect of the propolis extracts was evaluated according to the method described by Campos et al. [15], with minor modifications. The assays were conducted with erythrocyte suspensions. The erythrocytes were preincubated at 37C for 30 min in the presence of different concentrations of ascorbic acid or ExEP (50–125 μg/mL). Then, 50 mM 2,2’-azobis-(2-amidinopropane) dihydrochloride (AAPH) solution was added. Total hemolysis was induced by incubating erythrocytes with distilled water. Basal hemolysis caused by ExEP was assessed by incubating erythrocytes with the extract without the presence of AAPH, and the negative controls were assessed in erythrocytes incubated with 0.9% NaCl or 1% ethanol. This mixture was incubated at 37^°^C, with periodical stirring. Hemolysis was determined after 120, 180 and 240 min of sample incubation; specifically, samples were centrifuged at 1500 rpm for 10 min and aliquots of there were transferred to tubes with 0.9% NaCl, after which the absorbance of the supernatant was read spectrophotometrically at 540 nm. The percentage hemolysis was measured with the formula A/B × 100, where (A) is the sample absorbance and (B) is the total hemolysis. Three independent experiments were performed in duplicate.

#### Dosage of malondialdehyde (MDA)

The inhibition of lipid peroxidation was determined by the quantification of the levels of malondialdehyde, a marker of oxidative damage of the membrane lipids. For this, 10% erythrocyte suspension was used to assess the protective effects of ExEP against lipid peroxidation, evaluated through the dosage of malondialdehyde (MDA), as described by Campos et al. [16], with some modifications. Erythrocytes were preincubated at 37°C for 30 min with different concentrations of ascorbic acid or ExEP (50–125 μg/mL). The negative controls were assessed in erythrocytes incubated with 0.9% NaCl or 1% ethanol. Next, 50 mM AAPH was added to the erythrocyte solution, which was then incubated at 37°C for 4 hours with periodical stirring. At 120, 180 and 240 min of incubation, the samples were centrifuged at 1500 rpm for 10 min, and 500 μL aliquots of the supernatant were transferred to tubes with 1 mL of 10 nmol thiobarbituric acid (TBA), dissolved in 75 mM monobasic potassium phosphate buffer at pH 2.5. As a standard control, 500 μL of 20 mM MDA solution were added to 1 mL of TBA. The samples were incubated at 96°C for 45 min. The samples were then cooled, 4 mL of n-butyl alcohol were added and the samples were centrifuged at 1500 rpm for 10 min. The absorbance of supernatants sample was read at 532 nm. Three independent experiments were performed in duplicate. MDA levels in the samples were expressed in nmol/mL, obtained with the following formula 2:

MDA=Abssamplex(20x220.32/Absstandard) (2)

### Cell line and culture conditions

Human peripheral blood mononuclear cells from healthy donors were collected after informed patient consent. Separation of mononuclear cells was performed by gradient centrifugation methods using Ficoll Histopaque-1077 (1.077 g/cm^3^) (Sigma–Aldrich, Germany) follow the manufacturer’s instructions at 400 x g for 30 min. The use of human samples was approved by the local Ethical Committee of the University Center of Grande Dourados under protocol number 123/12. The K562 human cell line derived by chronic myelogenous leukemia was grown is suspension in RPMI 1640 media (Cultilab, Campinas, São Paulo, Brazil) supplemented with 10% fetal bovine serum (FBS; Cultilab), 100 U/mL of penicillin (Sigma-Aldrich, Germany) and 100 μg/mL of streptomycin (Sigma-Aldrich, Germany) in a humidified atmosphere at 37°C in 5% CO_2_.

### Cytotoxic activity and cell death profile

The cytotoxicity and possible mechanisms of death promoted by ExEP were determined by cytotoxic activity and cell death profile, evaluated according to the method described by Paredes-Gamero et al. [34], with minor modifications. Peripheral blood mononuclear cells and K562 cells were seeded into 96-well plates (5 x10^5^ cell/well) and cultured in medium with 10% FBS for 24 h with different concentrations (0.0625–1 mg/mL) or IC_50_ of ExEP-P (0.38 mg/mL) or ExEP-A (0.36 mg/mL). For dilution of the highest concentration of extract was used 0.2% ethanol, which was tested as a negative control (data not shown). All other concentrations were diluted only in culture medium. The positive control was only culture medium. After this period, the K562 cells were washed with PBS and resuspended in annexin-labeling buffer (0.01 M HEPES, pH 7.4, 0.14 M NaCl and 2.5 mM CaCl_2_). The suspensions were stained with annexin-FITC and propidium iodide (PI) (Becton Dickinson, Franklin Lakes, NJ, USA), according to the manufacture’s instructions. The cells were incubated at room temperature for 15 min. Three thousand events were collected per sample, and the analyses were performed on a FACSCalibur flow cytometer (Becton Dickinson) with CellQuest software (Becton Dickinson).

### Cell viability

K562 cells were seeded at 5 x 10^5^ cells/mL in 12-well microplates and treated with IC_50_ of the ExEP-P or ExEP-A and incubated for 48 h. The cells were observed after 0, 4, 8, 24 and 48 h of incubation using an inverted microscope under 10X objective (Nikon Eclipse TS 100) connected to digital camera (Nikon DS-1).

### Caspase-3 activity

Caspase-3 activity was measured by flow cytometer according to the method described by Moraes et al. [35]. K562 erythroleukemia cells were treated with IC_50_ of the ExEP-P or ExEP-A in 24-well microplates (5 x 10^5^ cells/mL) for 4, 8, 24 and 48 h. Then the cells were fixed with 2% paraformaldehyde in PBS for 30 min and permeabilized with 0.01% saponin for 15 min at room temperature. Next, the cells were incubated for 1 h at 37^∘^C with anti-cleaved-caspase 3-FITC antibody (Becton Dickinson, USA). After incubation for 40 min, the fluorescence was analyzed by Accuri C6 flow cytometer (Becton Dickinson, USA). A total of 10,000 events were acquired. Alternations in the fluorescence intensity were determined by comparing the levels of the treated cells to those of the controls.

### Effect of inhibitors on ExEP-induced cell death

K562 cells were seeded in 96-well microplates (5 x 10^5^ cells/mL) containing RPMI 1640 supplemented with 10% FBS in the presence of 20 μM of necrosis inhibitor necrostatin-1 (NEC-1), and they were incubated in a humidified atmosphere at 37°C and 5% CO_2_ for 60 min. Afterwards, the IC_50_ of the ExEP-P or ExEP-A were added to each sample, and the mixture was incubated for 24 h. Then, the cells were washed with PBS, resuspended in Annexin buffer (0.01 M HEPES, pH 7.4, 0.14 M NaCl and 2.5 mM CaCl_2_) and incubated for 20 min at room temperature after the addition of annexin V-FITC and propidium iodide (Becton Dickinson, Franklin Lakes, NJ) according to the manufacturer's instructions. The analyses were performed using an Accuri C6 flow cytometer (Becton Dickinson) and Accuri C6 software (Becton Dickinson), with 4000 events collected per sample.

### Statistical analyses

All data are represented by the mean ± standard error of the mean (SEM), based on at least two independent experiments. To establish the half-maximal inhibitory concentration (IC_50_) of DPPH free radical scavenging, the samples were tested in serial dilutions (1, 10, 50, 100, 200, 300 and 500 μg/mL) and analyzed by means of nonlinear regression using the Prism 6 GraphPad Software. The significant differences between the different groups were evaluated by analysis of variance (ANOVA) followed by Dunnett's post-test, using the GraphPad prism 6 program. The results were considered significant when p < 0.05.

## Results

### Chemical composition

The propolis extracts produced by different bee species had a similar chemical profile (Tables 1 and 2 and S1 Fig), although some compounds were identified exclusively in ExEP-A. The major compounds identified in ExEP-P were tocopherol, β-amyrin, ferulic acid and β-amyrin acetate, and the major compounds in ExEP-A were cinnamic acid, tocopherol, β-amyrin and apigenin. Both extracts presented similar amounts of *p*-methylbenzoic acid and caffeic acid, but ExEP-P contains higher amounts of amyrin, tocopherol, vanillin and ferulic acid analogues. In contrast, ExEP-A presented approximately 2.5 times more cinnamic acid and 2 times more *p*-coumaric acid and exclusively the compounds apigenin, luteolin, rutin, sinapic acid, α-amyrin acetate, taraxasterol, campesterol and stigmasterol.

**Table 1:** Compounds identified in nonpolar fraction of ExEP from *P*. *droryana* (ExEP-P) and *A*. *mellifera* (ExEP-A) by gas chromatography−mass spectrometry (GC-MS).

Peak	Retention time (min)	Compounds	Molecular mass	ExEP-P mg/g ± SD	ExEP-A mg/g ± SD
1	16.46	campesterol	400	-	7.2 ± 0.2
2	17.02	stigmasterol^a^	412	-	2.4 ± 0.1
3	17.72	β-sitosterol^a^	414	7.2 ± 0.3	3.3 ± 0.2
4	17.93	β-amyrin^a^	426	14.5 ± 0.5	11.3 ± 0.4
5	18.09	taraxasterol	426	-	3.6 ± 0.1
6	18.45	α-amyrin^a^	426	8.8 ± 0.2	3.8 ± 0.1
7	19.65	β-amyrin acetate^a^	468	12.2 ± 0.4	8.1 ± 0.2
8	21.20	α-amyrin acetate^a^	468	-	6.4 ± 0.3
9	24.56	tocopherol^a^	430	16.8 ± 0.6	13.3 ± 0.5

This is the Table 1 legend.

^a^Compound confirmed by comparison with standard

Data are shown as mean ± standard deviation

*n* = number of independent experiments in triplicate

**Table 2:** Compounds identified in polar fraction of ExEP from *Plebeia droryana* (ExEP-P) and *Apis mellifera* (ExEP-A) by high-performance liquid chromatography (HPLC).

Peak	Retention time (min)	Compounds	ExEP-P mg/g ± SD	ExEP-A mg/g ± SD
1	8.64	caffeic acid	5.3 ± 0.2	4.9 ± 0.2
2	10.44	vanillin	5.6 ± 0.2	-
3	13.48	*p*-coumaric acid	2.7 ± 0.1	5.2 ± 0.2
4	17.28	ferulic acid	14.1 ± 0.3	3.8 ± 0.1
5	19.99	*p*-methylbenzoic acid	7.6 ± 0.3	7.1 ± 0.3
6	21.97	sinapic acid	-	1.8 ± 0.1
7	25.10	rutin	-	5.1 ± 0.2
8	35.33	quercetin	5.7 ± 0.2	6.9 ± 0.3
9	36.68	luteolin	-	3.3 ± 0.1
10	40.01	cinnamic acid	6.5 ± 0.2	16.4 ± 0.5
11	42.62	apigenin	-	8.6 ± 0.3

This is the Table 2 legend.

Data are shown as mean ± standard deviation

*n* = number of independent experiments in triplicate

### Free radical-scavenger activity

Both ExEP presented antioxidant activity against the DPPH free radical. ExEP-P was able to inhibit 50% of free radicals (IC_50_) at a concentration of 182.4 ± 58.9 μg/mL and had a maximum activity of 94.6 ± 0.9% of DPPH radical capture at 500 μg/mL, being approximately 3.7 times less efficient than ExEP-A, which presented an IC_50_ of 49.8 ± 4.99 μg/mL and a maximum activity of 94.6 ± 0.3% at 300 μg/mL (Table 3). ExEP-A showed similar antioxidant activity as the synthetic antioxidant BHT, which presented IC_50_ of 52.8 ± 19.3 μg/mL and a maximum activity of 93.5 ± 0.5% at 500 μg/mL. The antioxidant standard ascorbic acid showed IC_50_ of 3.16 ± 0.6 μg/mL and maximum activity of 96.8 ± 0.4% at 10 μg/mL (S2 Fig).

**Table 3:** DPPH free radical-scavenging activity (%) of ExEP at different concentrations (μg/mL).

Sample	Concentration (μg/mL)							
	1	10	50	100	200	300	500	IC_50_
**Asc. acid**	12.8 ± 2.7	93.7 ± 0.5	96.8 ± 0.4	96.5 ± 0.6	96.7 ± 0.4	97.0 ± 0.4	97.0 ± 0.3	3.36 ± 0.8
**BHT**	4.1 ± 1.7	20.4 ± 3.4	54.5 ± 5.9	71.9 ± 4.1	86.5 ± 1.7	90.1 ± 0.5	93.8 ± 0.4	52.8 ± 19.3
**ExEP-P**	23.4 ± 2.9	21.2 ± 0.9	29.9 ± 1.8	44.3 ± 3.1	57.8 ± 1.0	74.3 ± 2.2	94.6 ± 0.9	182.4 ± 58.9
**ExEP-A**	4.5 ± 1.6	16.0 ± 1.5	47.8 ± 2.2	80.0 ± 1.3	90.4 ± 0.4	94.6 ± 0.3	92.7 ± 0.6	49.8 ± 5.0

Asc. acid = Ascorbic acid

### Oxidative hemolysis inhibition assay

**Fig 1:**
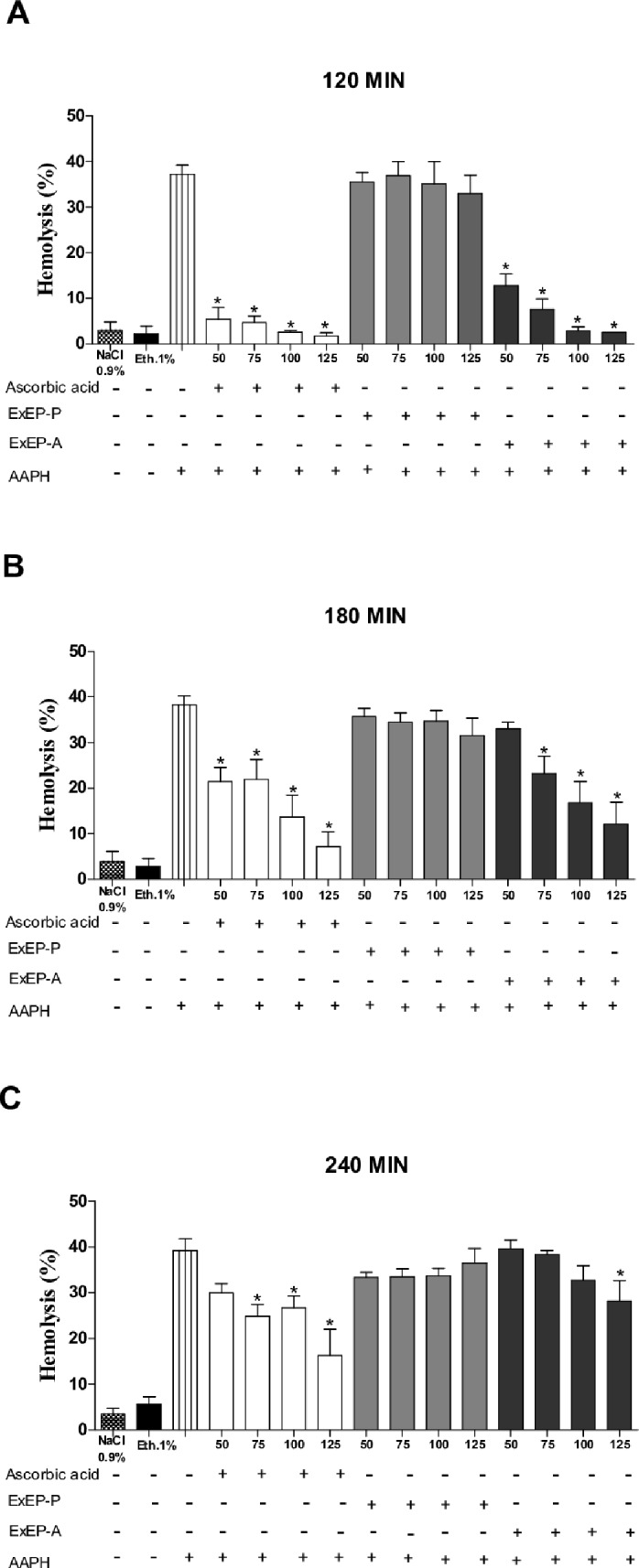
Protective effect of ascorbic acid (standard antioxidant) and ethanolic extracts of *P*. *droryana* (ExEP-P) and *A*. *mellifera* (ExEP-A) propolis against hemolysis induced by AAPH in human erythrocyte suspension at (A) 120 (B) 180 and (C) 240 min evaluation. NaCl (0.9%) and 1% ethanol was employed as negative controls. The results are expressed as mean ± SEM (standard error of the mean), n = 3. *Significantly different (p < 0.05) compared to the AAPH control group. With the exception of the negative control, all treatments were incubated with the oxidizing agent AAPH.

The antioxidant activity was also evaluated by an inhibition assay against AAPH-induced hemolysis. ExEP-P was not able to inhibit hemolysis induced by the oxidizing agent AAPH. The ascorbic acid control and ExEP-A showed concentration- and time-dependent anti-hemolytic activity. Ascorbic acid and ExEP-A inhibited 52.9 ± 15.6% and 24.6 ± 12.3% of hemolysis compared to the AAPH control, respectively, at a concentration of 125 μg/mL, after 240 min of incubation (Fig 1). The ascorbic acid control and ExEP, in the different concentrations evaluated, did not show hemolytic action when incubated with red blood cells alone, in the absence of the oxidizing agent AAPH (S3 Fig).

### Dosage of malondialdehyde (MDA)

**Fig 2:**
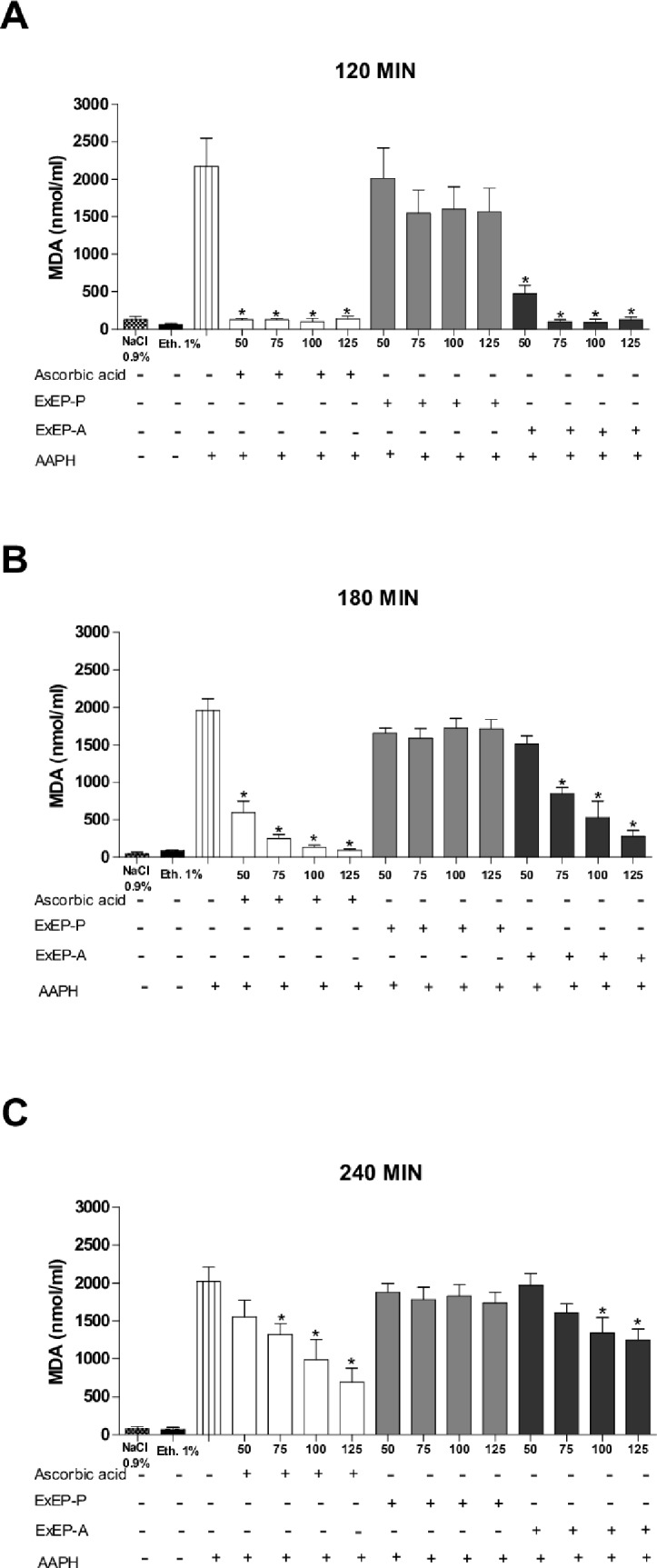
Concentrations of malondialdehyde (nmol/mL) after incubation of human erythrocytes with ascorbic acid (standard antioxidant) and ethanolic extracts of propolis from *P*. *droryana* (ExEP-P) and *A*. *mellifera* (ExEP-A), induced by oxidizing agent AAPH at (A) 120 (B) 180 and (C) 240 min of evaluation. NaCl (0.9%) and 1% ethanol was employed as negative controls. The results are expressed as mean ± SEM (standard error of the mean), n = 3. *Significantly different (p < 0.05) compared to the AAPH control group. With the exception of the negative control, all treatments were incubated with the oxidizing agent AAPH.

The efficiency of ExEP in inhibiting AAPH-induced lipid peroxidation was assessed based on its ability to reduce levels of MDA, a by-product of lipid peroxidation. ExEP-P was not able to inhibit the MDA content generated by the action of the oxidizing agent AAPH. The ascorbic acid control and ExEP-A reduced the levels of MDA by 65.7 ± 9.0 and 38.4 ± 7.3%, respectively, compared to the AAPH control, after 240 min of incubation at the highest concentration evaluated (Fig 2).

### Cytotoxic activity and cell death profile

**Fig 3:**
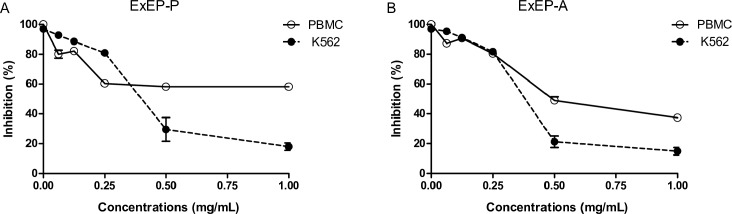
Cytotoxic activity of ExEP from (A) *P*. *droryana* and (B) *A*. *mellifera* against the peripheral blood mononuclear cells (PBMC) and K562 erythroleukemia cell lines.

**Fig 4:**
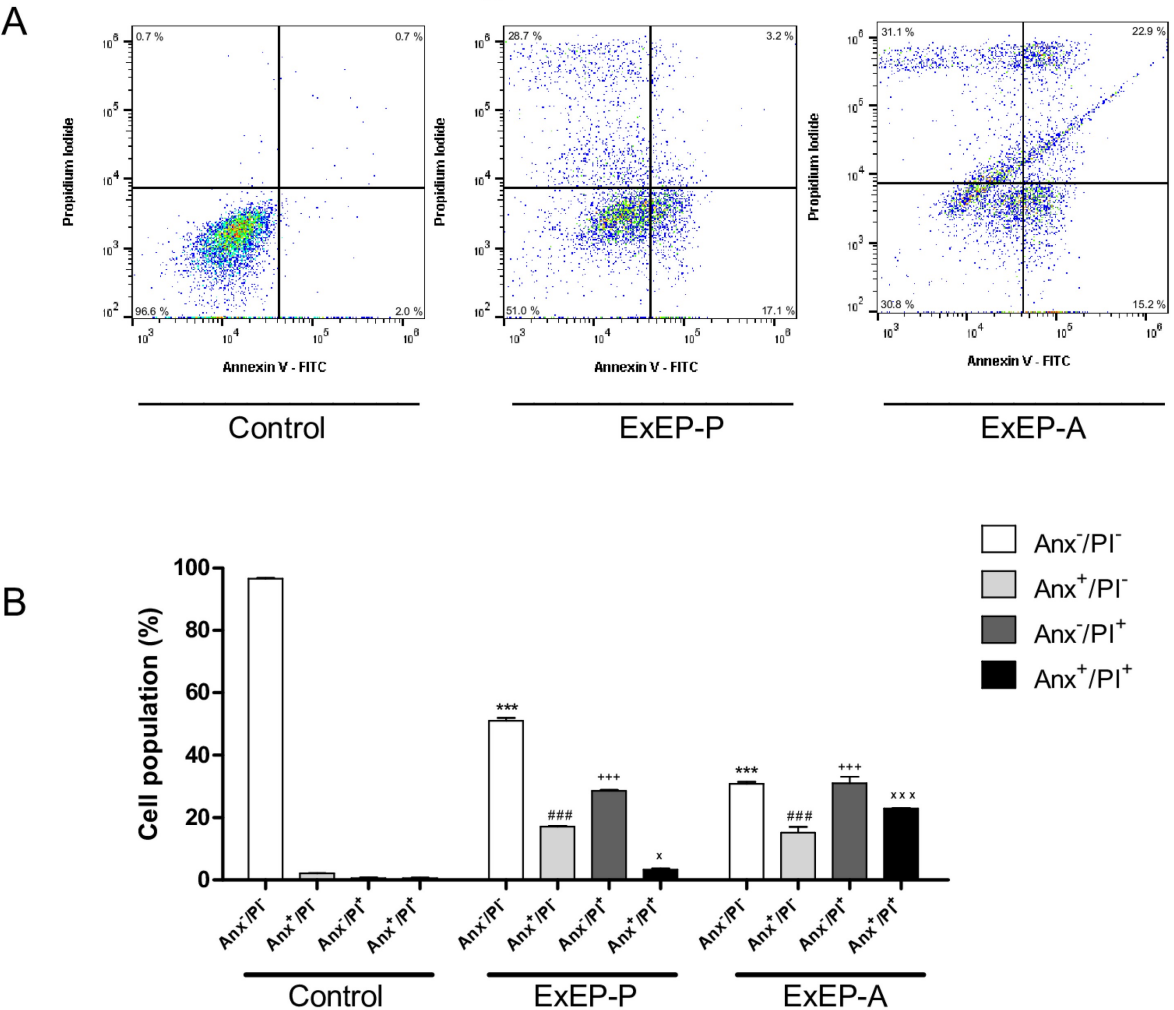
Cell death profile after treatment with IC_50_ of the ExEP-P and ExEP-A. (A) Dot plots indicating the flow cytometry, and (B) representative diagrams obtained via flow cytometry of cells stained with annexin V-FITC/PI; Anx^–^/PI^–^: viable cells; Anx^+^/PI^–^: apoptotic cells; Anx^–^/PI^+^: necrotic cells, and Anx^+^/PI^+^: cells in late apoptosis. ***p < 0.001 treated group versus control viable cells. ^###^p < 0.001 treated group versus control apoptosis. ^+++^p < 0.001 treated group versus control necrosis. ^xxx^p < 0.001 and ^x^p < 0.05 treated group versus control late apoptosis.

Peripheral blood mononuclear and K562 cells were treated with ExEP-P and ExEP-A to assess cell cytotoxicity. Both extracts of propolis showed lower cytotoxicity against peripheral blood mononuclear cells than K562 cells. The ExEP-P (IC_50_ = 0.38 mg/mL) and ExEP-A (IC_50_ = 0.36 mg/mL) promoted the cell death in K562 cells (Fig 3) after 24 h of treatment, the main mechanisms of death observed in both extracts was necrosis (Fig 4). The results show that propolis produced by different species of bees induce the same cell death mechanism.

### Effect of ExEP on the K562 cell viability

**Fig 5:**
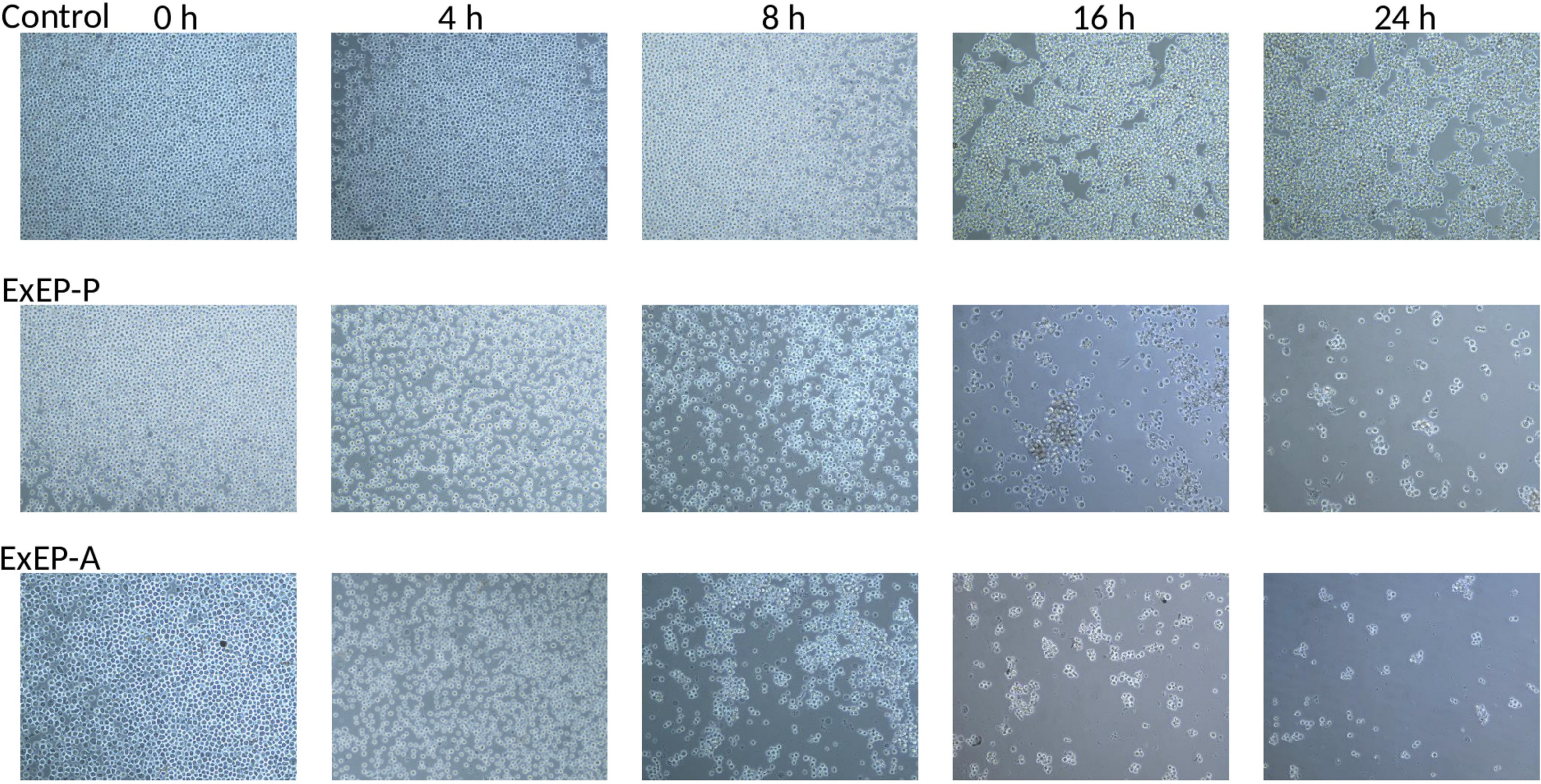
Viability of K562 cells at 0, 4, 8, 24 and 48 h after treatment with IC_50_ of the ExEP-P or ExEP-A. Images are representative of those seen from at least three such fields of view per sample and three independent repeats.

The viability of the K562 cells without and with treatment (IC_**50**_) of the ExEP-P or ExEP-A, was observed under an inverted microscope. No significant change was observed in control cells, however, for both ExEP, the cell survival decreased with increasing time (Fig 5). The results showed that the extracts of propolis were antiproliferative agents against K562 cells.

### Caspase-3 activity

**Fig 6:**
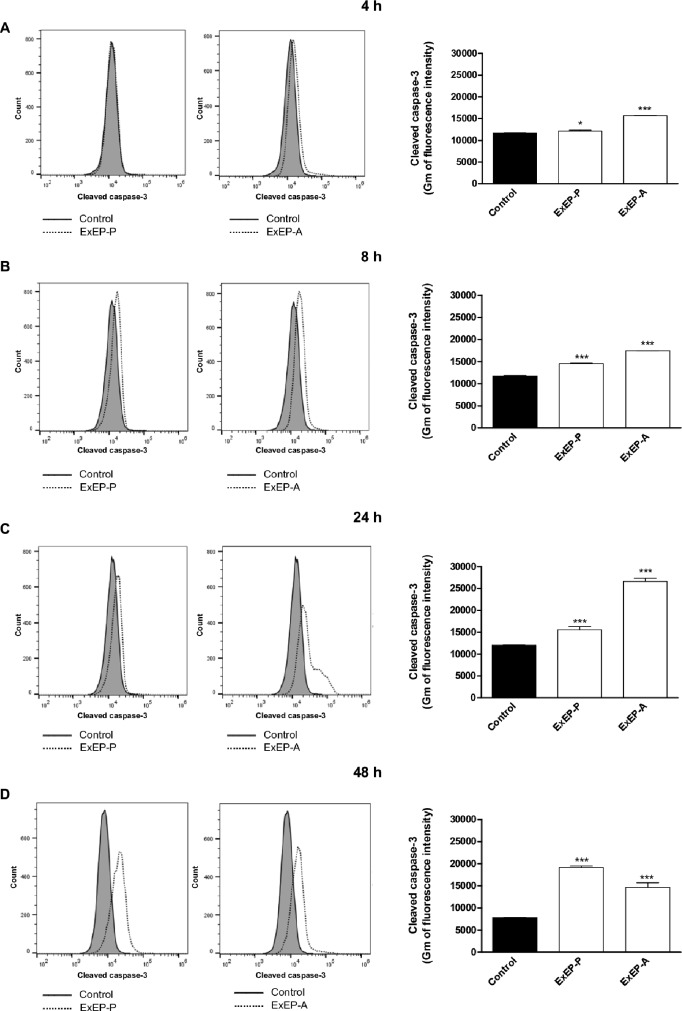
Caspase-3 activation in K562 cells treated with ExEP-P and ExEP-A after (A) 4, (B) 8, (C) 24 e (D) 48 hours. *p < 0.05, **p < 0.01, and ***p < 0.001 compared with the control group.

A monoclonal anti-cleaved caspase-3 antibody was used to evaluate caspase-3 activation in cells K562 incubated with IC_50_ of the ExEP-P and ExEP-A, and the cells were analyzed via flow cytometry. Both extracts resulted in the cleavage of procaspase 3 in 4, 8, 24 and 48 h, as indicated by a shift in fluorescence to the right (Fig 6) compared with the untreated control.

### Effect of inhibitors on ExEP-induced cell death

**Fig 7:**
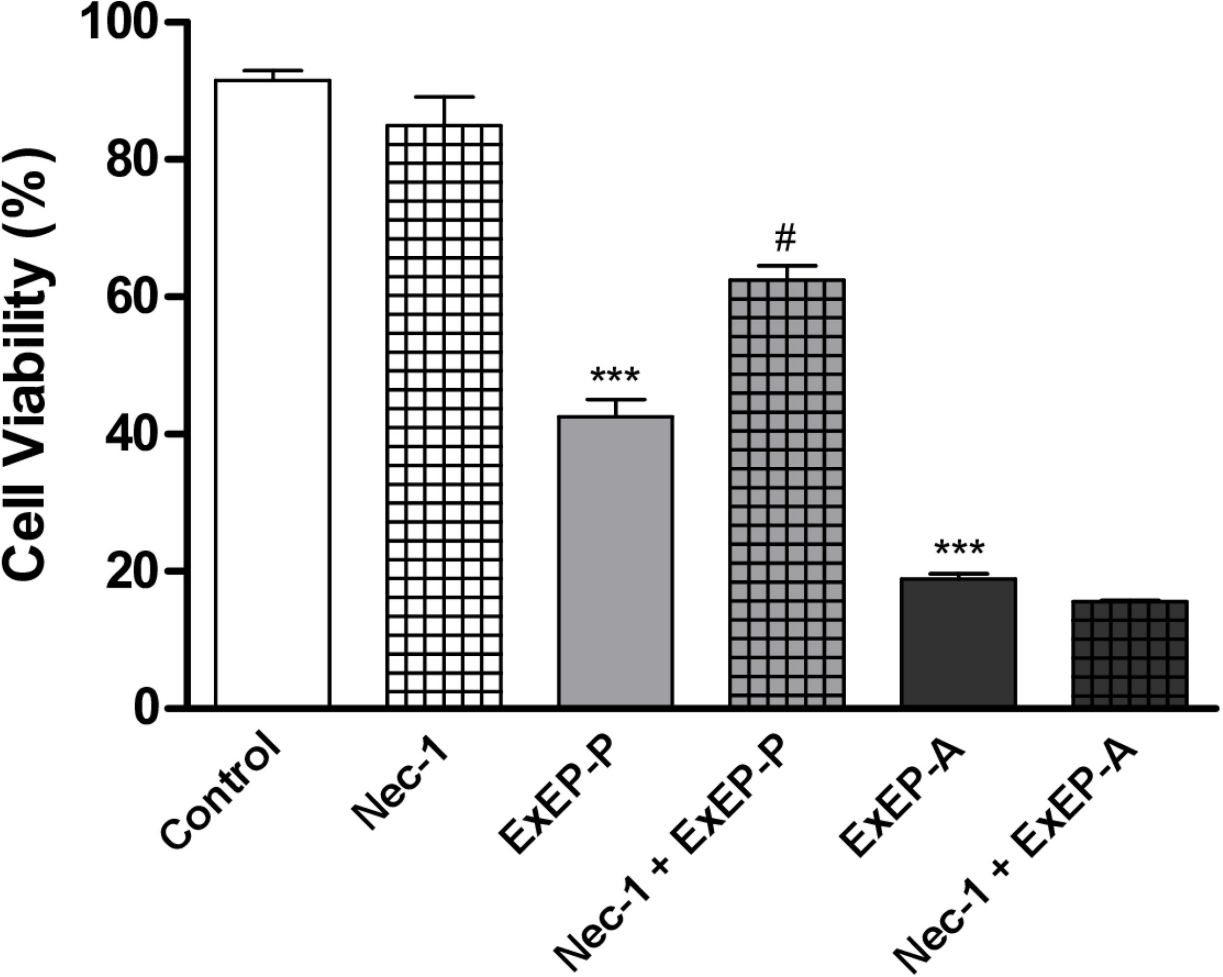
Effect of a necrosis inhibitor necrostatin-1 (NEC-1) on cell death mediated by ExEP-P and ExEP-A. *** p<0.0001 compared with the control group. # p<0.05 NEC-1+ ExEP-P compared with the ExEP-P.

The necrosis inhibitor necrostatin-1 (NEC-1) was effective in inhibiting the ExEP-P-induced death (IC_50_ = 0.38 mg/mL) of K562 cells treated for 24 h. However, the ExEP-A (IC_50_ = 0.36 mg/mL) was ineffective in inhibiting cell death (Fig 7).

## Discussion

Propolis is among the oldest natural products described for medicinal purposes and used by ancient societies. This bee product is a promising bioactive blend incorporating phenolic compounds, terpenes and tocopherol. These classes of compounds have already been identified in samples of propolis from different species of bees because they present anti-inflammatory [17–19], antimicrobial [14,34], antibiofilm [12,13], antioxidant [10,36] and antitumour effects [15,16,31,37].

In propolis from Brazilian meliponinae, phenolic compounds and triterpenes have been identified among the major constituents [27]. In addition, compounds such as cinnamic acid, coumaric acids and caffeates are among the main bioactive compounds of propolis [8,36,38–41] and have been described as potential antioxidants [32,36,42,43] due to their chemical structures [36,44].

The structures of phenolic compounds have at least one aromatic ring with one or more attached hydroxyl groups, which are capable of donating hydrogen or electrons, preventing the oxidation of other substances, particularly lipids [39,45]. Another mechanism by which these compounds exert antioxidant activity is via the inactivation of enzymes (xanthine oxidase, protein kinase C, ascorbic acid oxidase) involved in the production of reactive oxygen species (ROS) [36]. Thus, the phenolic compounds may be related to the antioxidant activity exerted by the ExEP of *P*. *droryana* and *A*. *mellifera*. The extracts of propolis evaluated were able to inhibit the DPPH free radical. In the body, ROS are produced during the cell cycle and functional activities, and they play important roles in various biological processes, such as cell signalling, apoptosis and gene expression [46–48].

However, excessive ROS production may result in oxidative stress, which is characterized by an imbalance between the production of oxidizing substances and endogenous antioxidants, and may cause the oxidation of biomolecules present in the cells, mitochondrial dysfunction and the activation of caspase cascades, resulting in cell death [47,49]. Oxidative stress has been found to be a trigger for chronic diseases such as diabetes, cardiovascular disease and cancer [40].

The antioxidant defense system of the human organism involves a set of enzymes such as superoxide dismutase (SOD), catalase (CAT), glutathione peroxidase (GPx) and glutathione reductase (GR), as well as, a set of non-enzymatic substances such as glutathione, composed of an active thiol group and acts in the elimination of reactive species and as cofactor for several antioxidant enzymes. Besides this, ascorbic acid, carotenoids, α-tocopherol, and other dietary antioxidants also act against damage induced by high concentrations of ROS. However, these protection systems are sometimes insufficient to completely prevent oxidative damage [41]. These factors demonstrate the importance of identifying natural compounds and/or new substances that can neutralize these free radicals to prevent oxidative stress.

In this study, the antioxidant activity of ExEP was also evaluated by testing its protection against oxidative hemolysis and the ability to reduce the levels of MDA, a product of lipid peroxidation due to oxidative stress [50]. The cell membrane is one of the structures most susceptible to the action of ROS due to lipid peroxidation, which causes changes in its structure and permeability [36,40].

However, unlike the results obtained by the direct DPPH free radical capture assay, only *A*. *mellifera* propolis extract was able to protect red blood cells against damage by the oxidizing agent AAPH. These results may be related to the chemical composition of this propolis, since ExEP-A presented higher amounts of cinnamic acid than ExEP-P, in addition to campesterol, stigmasterol, taraxasterol, rutin, luteolin and apigenin identified exclusively in ExEP-A. Rutin and apigenin, have already been described as antioxidant agents [51, 52], and may be related to the antioxidant activity of this extract.

Although ExEP-A showed higher antioxidant activity than ExEP-P, the cytotoxic activity of the both extracts was similar. This result suggests that compounds that promote antioxidant action may not be responsible for the antitumor action of the extracts. Thus, the compounds caffeic acid, quercetin and tocopherol described by their antitumor activities [53–55] were identified in both extracts and may be related to this biological activity of both extracts.

In addition, other compounds found in the extracts were also reported as cinnamic acid, rutin, apigenin and taraxasterol have also been reported as potential antitumour agents [51,52,56,57]. Studies have shown that apigenin is an important oncogenesis blocker [56].

In evaluating the cytotoxic activity of propolis, it was observed that the ExEP could reduce the cellular viability of leukemic cells (K562). Thus, the compounds present in ExEP may be relevant in inhibiting tumour cells. Apigenin has been reported to inhibit the growth of laryngeal carcinoma cells [56]. Taraxasterol showed antitumour activity in glioblastoma cells [57]. In addition, caffeic acid, considered the main constituent of propolis, has already been reported to exhibit cytotoxic action in human myeloid leukemia cells [37].

For both extracts, the main mechanism of death observed was necrosis. Other cytotoxicity studies of stingless bee propolis showed the same mechanism of killing against K562 cells [15,16]. Franchi Jr. et al. [58] found that extracts of green and red propolis produced by *Apis mellifera*, in the southeastern and northeastern regions of Brazil, respectively, were cytotoxic against erythroleukemic strains; however, they promoted cell death via apoptosis.

Although apoptosis is among the main mechanisms of action of the drugs currently on the market, some *ex vivo* studies show that propolis extracts present different responses against tumour cells, regarding the mechanism of cell death [58,59]. In this context, necrosis-induced cell death may be an alternative for the treatment of tumour lines that show resistance to death by apoptosis.

In summary, our results show for the first time that the propolis produced by *P*. *droryana* and *A*. *mellifera* from the Brazilian Cerrado present potential use in the pharmaceutical and food industries, considering their antioxidant and cytotoxic properties against erythroleukemia cells.

## Supporting information

S1 Fig Chromatogram by GC-MS of the ExEP (A) *P*. *droryana*, (B) *A*. *mellifera*, and HPLC of the ExEP (C) *P*. *droryana* and (D) *A*. *mellifera*.
(EPS)

S2 Fig Nonlinear regression to establish the half-maximal inhibitory concentration (IC_50_) of DPPH free radical scavenging for ascorbic acid, BHT, ethanolic extracts of propolis of *P*. *droryana* and *A*. *mellifera*. (EPS)

S3 Fig Effect of ascorbic acid (standard antioxidant) and ethanolic extracts of *P*. *droryana* (ExEP-P) and *A*. *mellifera* (ExEP-A) propolis in human erythrocyte suspension at (A) 120 (B) 180 and (C) 240 min evaluation. NaCl (0.9%) and 1% ethanol was employed as negative controls. The results are expressed as mean ± SEM (standard error of the mean), n = 3. *Significantly different (p < 0.05) compared to the NaCl (0.9%) control group.
(EPS)
